# Swelling Behaviour of Superabsorbent Polymers for Soil Amendment under Different Loads

**DOI:** 10.3390/polym10030271

**Published:** 2018-03-06

**Authors:** Krzysztof Lejcuś, Michał Śpitalniak, Jolanta Dąbrowska

**Affiliations:** Institute of Environmental Engineering, Wrocław University of Environmental and Life Sciences, 50-363 Wroclaw, Poland; michal.spitalniak@upwr.edu.pl (M.Ś.); jolanta.dabrowska@upwr.edu.pl (J.D.)

**Keywords:** polymers, hydrogels, water absorbing geocomposite, absorbency under load, swelling rate

## Abstract

One of the most important among the numerous applications of superabsorbent polymers (SAPs), also known as hydrogels, is soil improvement and supporting plant vegetation in agriculture and environmental engineering. Currently, when water scarcity involves water stress, they are becoming still more commonly used for water retention in soil. As it turns out, one of the major factors influencing the superabsorbent polymers water retention capacity (WRC) is the load of soil. The study presents test results of absorbency under load (AUL) of SAPs. The object of the analysis was cross-linked copolymer of acrylamide and potassium acrylate, of a granulation of 0.50–3.15 mm. The authors analysed the water absorption capacity of the superabsorbent polymers under loads characteristic for 3 different densities of soil (1.3 g∙cm^−3^, 0.9 g∙cm^−3^, 0.5 g∙cm^−3^) and three different depths of application (10 cm, 20 cm, and 30 cm). Soil load and bulk densities were simulated by using weights. The experiments were conducted with a Mecmesin Multitest 2.5-xt apparatus. The obtained results demonstrate a very significant reduction in water absorption capacity by SAP under load. For a 30 cm deep layer of soil of bulk density of 1.3 g∙cm^−3^, after 1 h, this value amounted to 5.0 g∙g^−1^, and for the control sample without load, this value amounted to more than 200 g∙g^−1^. For the lowest load in the experiment, which was 0.49 kPa (10 cm deep layer of soil of a bulk density of 0.5 g∙cm^−3^), this value was 33.0 g∙g^−1^ after 60 min. Loads do not only limit the volume of the swelling superabsorbent polymer but they also prolong the swelling time. The soil load caused a decrease in the absorption capacity from 338.5 g∙g^−1^ to 19.3 g∙g^−1^, as well as a prolongation of the swelling time. The rate parameter (time required to reach 63% of maximum absorption capacity) increased from 63 min for the control sample to more than 300 min for the largest analysed load of 3.83 kPa. The implications of soil load on superabsorbent polymer swelling are crucial for its usage and thus for the soil system. This knowledge might be employed for the more effective usage of superabsorbent polymers in agriculture and environmental engineering, in which they are commonly used to retain water and to support plant growth.

## 1. Introduction

Superabsorbent polymers (SAPs) or hydrogels are three-dimensional, cross-linked hydrophilic polymers with the ability to absorb large quantities of water even up to 1000 times of their dry weight [1,2,3]. In the last decades, they have been applied in various fields including medicine, industry, agriculture, civil engineering, soil and hygienic products [4,5,6,7,8,9,10,11,12], And recently as concrete additives [13,14]. In the soil they perform the function of a moisture buffer with the ability to stabilise plant water status; therefore, SAPs can lower plant drought stress [15,16,17]. They can also prevent fertilizer compounds and plant protection agents from being washed out from root zone groundwater [18,19]. Water absorbed by SAPs may be used by plants thanks to the suction forces of roots. Most plants are able to take up to 95% of the water retained within the superabsorbent polymer [20]. Mineral compounds dissolved in water limit the water retention capacity (WRC) of superabsorbent polymers significantly [21]. Monovalent ions reduce absorption by 75%, while bivalent and trivalent ions may reduce it even by 90% [22,23]. The strength of inhibition depends on the type of monomer, the concentration and valence of ions in the solvent, and the cross-linking density of SAP [2]. They are also subjected to solubilisation, internalisation, and incorporation or mineralisation by soil microorganisms [24,25]. One of the ways to increase absorption capacity and decrease sensitivity towards ions in the solution is to synthesise the acrylic polymer with clayey molecules in the polymerisation process [26,27,28].

The simplest method of using superabsorbent polymers or polyacrylamide (PAM) in agriculture is to apply them directly onto the soil. However, it was proved that such a manner of application leads to a decrease in the strength parameters of soils [29,30] and a reduction in their permeability. This method has many other negative implications [31,32,33]. Nevertheless, so far, superabsorbent polymers were mostly applied into the soil by direct mixing, injection, or by hydrosowing with seeds [34].

Regardless of the method used, after the introduction into soil the superabsorbent polymer is under load. Soil load significantly affects its absorption capacity. However, the literature does not provide a description of the influence of soil load on the operating of SAPs. The presented results of SAP’s absorption under load (AUL) either refer to the description of the basic properties of synthesised superabsorbent polymers or they are discussed in the context of the application of superabsorbent polymer in personal care products [35,36,37]. Such results are not representative of applications in agriculture or environmental engineering. The objective of our study was to analyse absorbency under load and swelling kinetics under the load of soil and soil substrates of various densities. The authors have used weights to simulate the adopted bulk density characteristic for sandy soils, gardening substrates, substrates for roof gardens, and topsoil on earth structures in order to demonstrate that the influence of AUL is essential for the functionality of SAPs.

## 2. Materials and Methods

### 2.1. Materials

To determine the influence of soil load on SAP, absorbency under load (AUL) was tested on cross-linked copolymer of acrylamide and potassium acrylate—Aquasorb 3005 KL (SNF FLOERGER, Andrézieux, France) that was available on the market. It is a commonly used material produced for agriculture and civil engineering, both in the aspect of its chemical composition and grain size distribution. SAP was delivered by the producer as air-dry granular material.

The grain size distribution of the superabsorbent polymer was determined with use of the sieve method. The 500 g sample of the material was sieved by pouring the dry material onto a sequence of 6 sieves with mesh widths of 0.25, 0.5, 1, 2, 5, and 10 mm. The tests were repeated 3 times and the results were averaged. As a result, a grain size distribution curve with range of grains and a grain size distribution histogram were created.

### 2.2. Measurment of AUL and Swelling Kinetics

The tests were based on the measurement concept developed by Ramazani-Harandi et al. [38]. Absorbency under load tests (AUL) was conducted in the Multitest 2.5-xt apparatus manufactured by Mecmesin.

**Figure 1:**
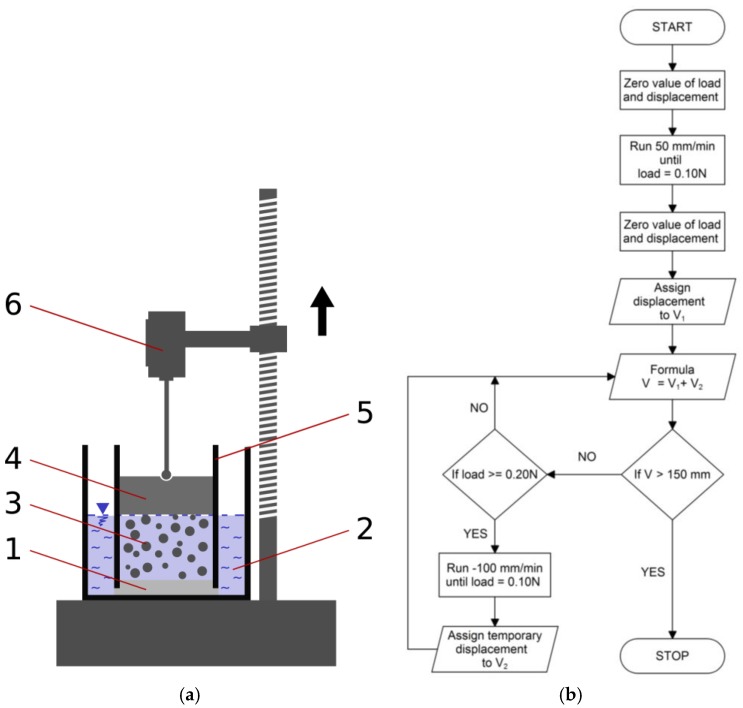
(**a**) Scheme of the test apparatus. (1) Porous rock, (2) outer cylinder with water, (3) superabsorbent polymer, (4) load, (5) inner cylinder, and (6) force sensor; (**b**) scheme of the test program procedure.

For the purposes of the investigation, a special set of two plexiglas cylinders, shown in Figure 1a, were prepared. The inner cylinder with sample and measuring sensor was placed inside the outer cylinder. The bottom of the inner cylinder was secured by porous rock of a thickness of 5 mm and a diameter of 72 mm. The amount of water in the outer cylinder was controlled in order to avoid water hydraulic pressure influence on the sensor device, and it was equal to the height of SAP sample in the inner cylinder throughout the duration of the tests. Distilled water of the temperature of 23 °C was used in the tests. A sample of SAP of a mass of 2 g was placed in the inner measurement cylinder and loaded with a weight corresponding to 10 cm, 20 cm, and 30 cm layer of soil of and bulk density of 0.5, 0.9, and 1.3 g∙cm^−3^. The range of the selected bulk density values reflects the application of superabsorbent polymers in sandy soils, gardening substrates, substrates for roof gardens, and topsoil on earth structures; the depth levels are characteristic for planting or sowing various types of plants—crop plants, turfs, ornamental plants, trees, and shrubs [39,40,41,42]. Load caused by the soil layer was calculated pursuant to the following Equation (1) [43]:

(1) σ=ρ·g·h 

in which *σ*—is the soil load [Pa], *ρ*—is the bulk density of the overlying soil [kg∙m^−3^], *g*—is the acceleration due to gravity [m∙s^−2^], and *h*—soil layer height [m].

The results were then converted into kPa. Soil load and bulk densities were simulated, and no real soil was used for the conducted experiments. To obtain required load, metal weights were used to avoid friction that would have occurred between the cylinder and the soil if the soil had been used for this experiment.

Measurements were taken with use of a force sensor (ICL) of an accuracy of ± 0.1%. During swelling under load, the SAP samples pushed up the ICL force sensor. If the force measured by sensor exceeded 0.2 N, the sensor device moved up until the measured force decreased to 0 N. The sensor measured force at a frequency of 10 Hz. This procedure allowed us to measure the time of swelling and the height of the swollen superabsorbent. Additionally, the same test procedure was repeated for a sample without load, as a control sample. The controlling software (Emperor™) delivered by Mecmesin was used and programmed so as to ensure the swelling of superabsorbent polymer at constant load. The programmed test procedure is presented in Figure 1b.

The measured increase in sample height was converted into the value of swelling—g∙g^−1^. A series of measurements without load were carried out in the same way. AUL was calculated according to following Equation (2) [38]:

(2) $$AUL=\frac{W_{2}-W_{1}}{W_{1}}$$

in which *AUL* [g∙g^−1^]—is the absorbency under load, and *W*_2_ [g] and *W*_1_ [g] are the weights of swollen and dry superabsorbent polymer, respectively.

Data were processed with use of a Voight-based model [44,45]. An important aspect of the operation of superabsorbent polymers is the kinetics of the absorption process. The diffusion process leads to the swelling of polymers. Several mechanisms for the kinetics of the diffusion process have been proposed, from the simplest Fickian diffusion to the viscoelastic kind [46]. As far as superabsorbent polymers are concerned, the spring and dashpot Voight-based viscoelastic model is best known and most often applied for use in modelling creep and relaxation [35,44,45].

Pursuant to the Voight-based model, the swelling rate of superabsorbent polymer can be described by Equation (3) [44,45]:

(3) $$S_{t}=S_{e}\left(1-e^{-\frac{t}{\tau }}\right)$$

in which *S*_t_ g∙g^−1^ is swelling at time *t*, *S*_e_ g∙g^−1^ is equilibrium swelling (power parameter, absorption capacity), *t* [min] is time for swelling, and *τ* [min] is the rate parameter (time required to reach 63% of the maximum absorption capacity).

Using the Voight-based model, the rate parameter (*τ*) and equilibrium swelling (*S*_e_) were calculated for various loads.

In order to check the statistical significance of the influence of load on water absorption by SAP, statistical analysis of data was conducted. The Levene’s test was used to assess the equality of variances. The Dunnett’s C test (post hoc group comparison) was applied to compare 9 experimental groups (with different loads) with a single control (without load). Curve fitting and data analysis was conducted with use of XLSTAT add-on for Excel and the software package Statistica v. 12.5.

## 3. Results

### 3.1. Grain Size Distribution of the Tested Superabsorbent Polymer

**Figure 2:**
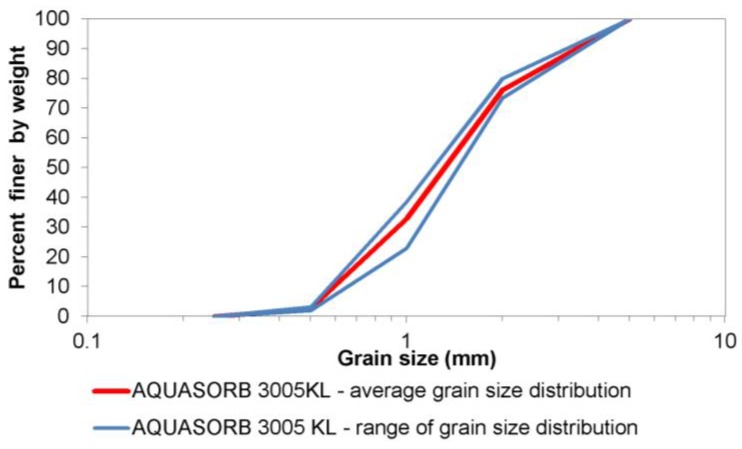
Grain size distribution of the tested superabsorbent polymer.

**Figure 3:**
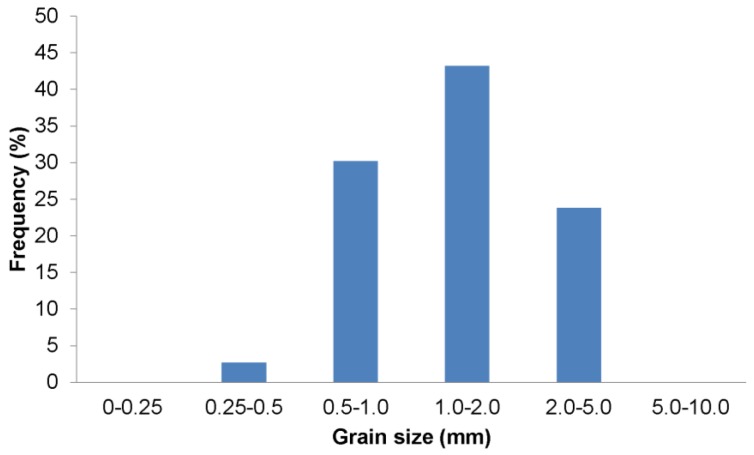
Histogram of grain size distribution by weight percent of fraction.

The amount and pace of absorption by the superabsorbent polymer do not depend only on its chemical structure and the properties of the solution where it occurs but also on its grain size distribution [22]. The grain size distribution of commercially available SAPs typically falls into the range of 0.1–4.0 mm. The grain size distribution of the analysed SAP is presented in Figure 2, and the histogram of grain size distribution is shown in Figure 3. The ranges with the highest share are the 1.0–2.0 mm range (over 43%) and the 0.5–1.0 mm range (which slightly exceeds 30%).

### 3.2. AUL and Swelling Kinetics

**Figure 4:**
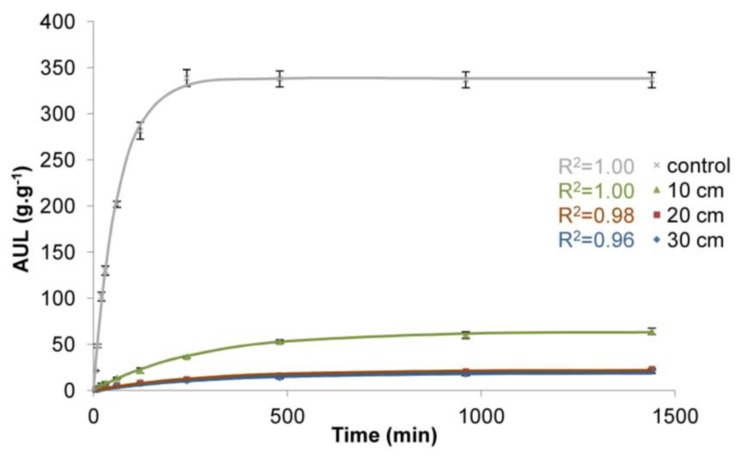
Time dependence of the AUL values for superabsorbent polymer loaded with soil of a bulk density of 1.3 g∙cm^−3^ and 10, 20, and 30 cm deep soil layers.

**Figure 5:**
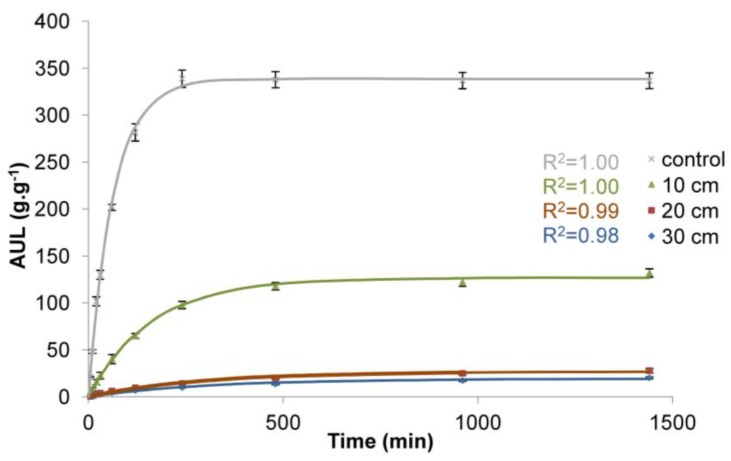
Time dependence of the AUL values for superabsorbent polymer loaded with soil of a bulk density of 0.9 g∙cm^−3^ and 10, 20, and 30 cm deep soil layers.

**Figure 6:**
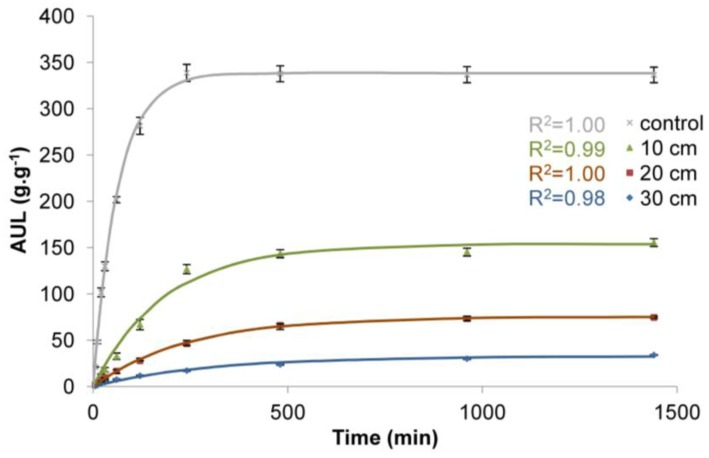
Time dependence of the AUL values for superabsorbent polymer loaded with soil of a bulk density of 0.5 g∙cm^−3^ and 10, 20, and 30 cm deep soil layers.

The obtained values of rate parameter (*τ*) and equilibrium swelling (*S*_e_) are presented in Table 1 and the adjusted curves are presented in Figure 4, Figure 5 and Figure 6.

The Levene’s test demonstrated that in the 10 analysed groups (9 groups of data for samples with load and 1 control group without load), variances were unequal. Due to that the Dunnett’s C test was applied in order to find significant differences between the groups, test has demonstrated that data series for samples under load are significantly different from the results obtained for the control group, and the calculated *p*-value was, in all cases, lower than the significance level 0.05. The results are presented in Table 2.

The results confirm the general influence of load on the functioning of superabsorbent polymers. Regardless of the load applied, the differences in water absorption are significant (Table 1, Figure 4, Figure 5 and Figure 6). The influence of load on water absorption of SAP is noticeable in each analysed case. The biggest differences with respect to control sample were obtained for the highest load. It amounted to 3.83 kPa, which corresponds to the load caused by a 30 cm deep layer of soil of a bulk density of 1.3 g·cm^−3^. This is a common value for sandy soils characterised by low water retention capacity, while the 30 cm deep layer of soil corresponds to the depth of a typical hole dug when planting larger bushes or smaller trees.

For the largest analysed load of 3.83 kPa, after the initial 10 min of measurement, the AUL was only 2 g∙g^−1^ compared to 48 g∙g^−1^ for the control sample (Figure 4). With the time, the AUL was still growing, although at a decreasing pace, until it finally reached the value of 21 g∙g^−1^ after 1440 min for the largest load, while in the case of the control sample the maximum value (339 g∙g^−1^) was obtained as early as after 120 min and then it fell slightly, to 337 g∙g^−1^ after 1440 min. It is noticeable that with the decreasing load (Figure 5), higher water absorption occurred. For the load of 1.77 kPa (20 cm deep layer of soil of a bulk density of 0.9 g∙cm^−3^), the AUL value after 60 min is 7 g∙g^−1^. However, even after 1440 min under such load, the SAP is not able to retain more than 28 g of water. Only for the lowest load in the experiment, which was 0.49 kPa, corresponding to 10 cm deep layer of soil of a bulk density of 0.5 g∙cm^−3^ (Figure 6), these values became significantly higher, 33 after 60 min and after 1440 min the value of 156 g∙g^−1^ was reached. However, this is still only 42% of the value for the control sample. Moreover, this result was obtained for very low bulk density of soil and a thin layer.

**Figure 7:**
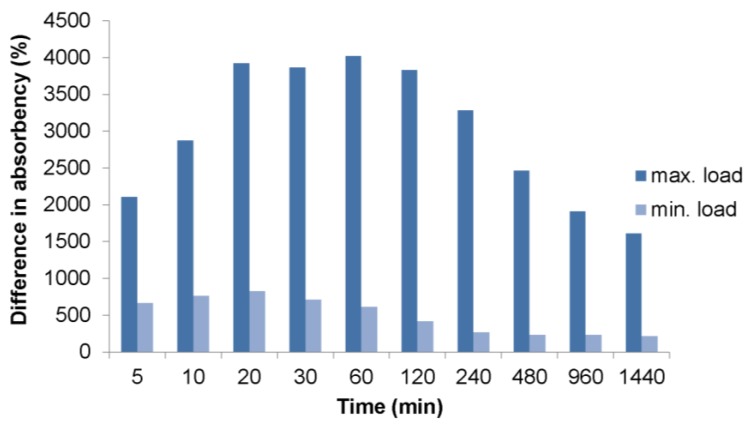
Percentage difference in water absorption.

Load has a very significant influence on water absorption by SAP. It turns out to be essential for the efficiency and reasonableness of the application of superabsorbent polymers in scattered form by means of mixing them with soil. The percentage differences in AUL under load and samples without load reach up to 4000% (Figure 7). The differences significantly influence the efficiency of the direct application of superabsorbent polymers into the soil, because much more SAP must be used to get the same amount of absorbed water compared to conditions without soil load.

**Figure 8:**
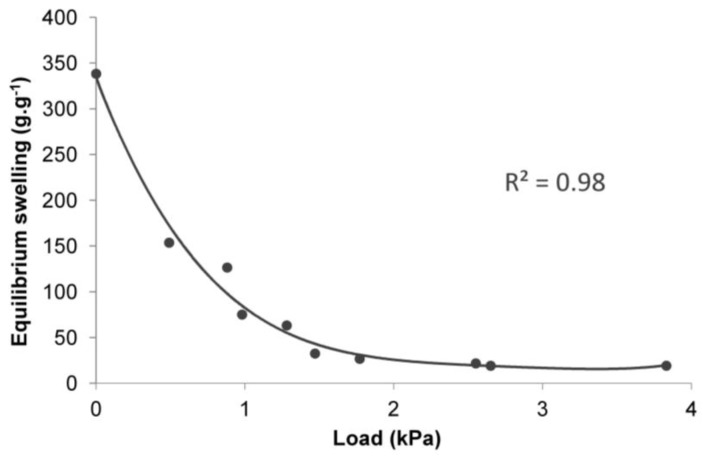
The dependence between equilibrium swelling (*S*_e_) and the load for all test groups and the control.

**Figure 9:**
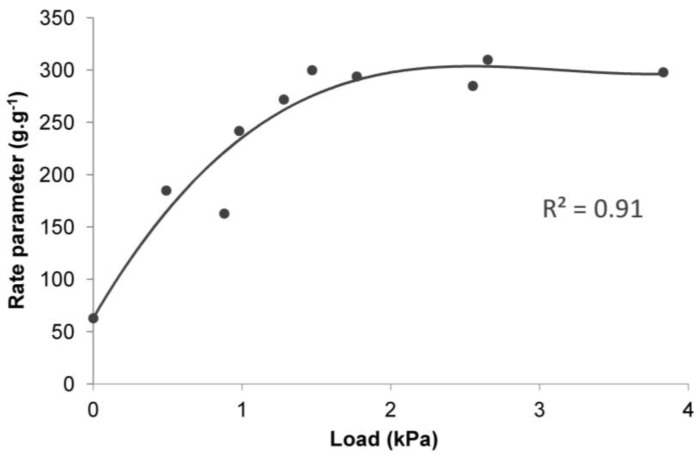
The dependence between the swelling rate parameter (*τ*) and the load for all test groups and the control.

The analysis of the obtained results of the adjustment of curves, compared to the results of laboratory tests (Table 1, Figure 4, Figure 5 and Figure 6), shows that the data fit well to a Voight-based model. Without load, the superabsorbent polymer reached 63% of *S_e_* = 339 g∙g^−1^ in 63 min, while for higher loads this time extended to 300 min, and the *S*_e_ fell to 19 g∙g^−1^ (Table 1). Loads do not only limit the volume of the swelling superabsorbent polymer, but they also prolong the swelling time. It is assumed that high swelling rate is required, as well as a high swelling capacity [47]. Figure 8 and Figure 9 lead to the same conclusion. The extension of swelling time in superabsorbent polymers that support plant vegetation may additionally lead to deterioration in their efficiency. In agriculture and environmental engineering, including erosion control, superabsorbent polymers are applied mainly in the case of sandy soils, characterised by a high infiltration rate [48]. In such soils, infiltration takes place very quickly, and the velocity of the wetting front in sandy soils is 5 times higher than in clayey soils [49,50]. If the water is not retained fast, most of it is lost for the plants.

For the purposes of the application of SAPs in agriculture or environmental engineering, the essential parameter is their capacity to retain water for later use by plants. The obtained results demonstrate that, regardless of the applied load that corresponds to various soil densities and various depths of soil layers, the differences between free absorption and absorption under load are extremely high. They may reach up to several hundred percent, even at the lowest applied load characteristic only for light horticultural substrates. These differences are particularly visible during the first two hours of measurement. For the highest applied loads, these differences reach 4000% and exceed 2000% for most of the measurement period. Both for maximum and minimum loads, the highest differences occurred between the 20th minute and 2 h of measurement time.

## 4. Discussion

Obviously, the results of these tests cannot be transferred directly to field conditions, which use superabsorbent polymer mixed with soil at various depths, in which the superabsorbent polymer may be inside soil pores or where there is only a small amount of empty space, etc. However, the obtained results confirm the significance of soil load in the case of application of superabsorbent polymers for engineering and agricultural purposes. The solution of the problem presented above may be a new type of geocomposite, which enables free water absorption by the superabsorbent inside the soil. The new water-absorbing geocomposite is free from the flaws of directly applied superabsorbent polymers and can limit water loss from soil [51].

It is difficult to compare the obtained results with the results obtained in tests on soils or horticultural substrates with the addition of SAPs. This results from the large variety of applied research methodologies and determined parameters. Typically, superabsorbent polymer additions to sandy soils 0.1, 0.2, 0.3, and 0.4% are tested [52,53,54]; in extreme cases they may reach up to 2% [55]. Tests were conducted on-site or in laboratory conditions, in cylinders placed in pots, or directly in the soil. The samples were irrigated or soaked in water for longer periods of time. The results were presented in form of volume or mass moisture content or field water capacity with respect to the control sample without superabsorbent in field capacity conditions. Additionally, measurements were taken at different times (several hours to several weeks after irrigation/soaking in water), and various types of SAPs were used in the analyses. Unfortunately, in most cases the authors do not specify the depth or thickness of the layer into which the SAP was applied, which is important with respect to the results presented in this study. The obtained results demonstrate a positive influence of the SAP addition on the moisture content of sandy soils (Table 3). In certain discussed cases, the results did not differ from the control sample in a statistically significant way [56]. Field tests conducted on several soil depth levels demonstrated that in the superficial layer the superabsorbent polymers noticeably improved the moisture conditions in comparison to the control sample, while in the subsequent layers the moisture in the control sample was significantly higher [15,57].

The results for 0.2% SAP addition to sandy soils showed an increase in volume moisture of 4–14% and in mass moisture 8–11% (Table 3). The results obtained by other authors may be compared with our laboratory test results by assuming that the SAP (0.2%) was applied through direct mixing on a given depth with sandy soil of a bulk density of 1.3 g∙cm^−3^; therefore, the potential increase in moisture content may be calculated. 24 h contact with water results in the increase in volume moisture by 3% for application at 30 cm depth, 6% for 20 cm, and 17% for application at 10 cm depth. If we convert these values into mass moisture content, we will obtain, respectively, 2%, 5%, and 13%. These outcomes are similar to those obtained by other authors. This confirms the thesis of the significance of AUL for the determination of the capacity of SAPs to improve soil retention. The conducted tests should take into account not only the type of soil and doses of SAP, but also the depth of application and soil density, as these factors determine the load imposed on the SAP when they absorb the water solutions in soil.

## 5. Conclusions

Absorbency under load (AUL) is an important parameter that determines the possibility to effectively apply superabsorbent polymers in environmental engineering and agriculture. The depth of application and bulk density of soil are some of the basic parameters that determine the extent of the limitation of water absorption by the SAP in soil. The comparison of the obtained test results with the field and laboratory studies based on direct application of SAP into soil show that the effectiveness of such application may be estimated based on the knowledge AUL of SAPs.

In the conducted laboratory tests, the differences in the amount of water retained (absorbed) by the superabsorbent polymer, in comparison to the control sample, for the analysed maximum load 3.83 kPa exceed 4000%. After 1 h, the results were, respectively, 5 g∙g^−1^ and 202 g∙g^−1^. Simulated soil load does not only limit the water absorption by the superabsorbent polymer, but it also extends the swelling time. The obtained results point to the necessity of reconsidering the different ways of applying SAPs to soil. One of the solutions may be to use water absorbing geocomposites or other forms of superabsorbent polymers that eliminate or minimise the influence of load on the water absorption process. For conducted studies, the depth of the application of SAP should be provided as one of the factors that determine the parameters of the experiment.

## 6. Patents

Presented results were essential to prepare a patent: “Geocomposite element, particularly for enhancing plant growth”, EP2560472, PL211198, which was commercialised under license.

## Figures and Tables

**Table 1:** Rate parameter (*τ*) and equilibrium swelling (*S*_e_) for the tested superabsorbent polymer.

Load (Simulated Soil Layer Depth and Bulk Density)	*τ* (min)	*S_e_* (g∙g^−1^)	Standard Error of the Regression (g∙g^−1^)	Coefficient of Determination R^2^ (-)	Load (kPa)
30 cm, *ρ* = 1.3 g∙cm^−3^	298	19.3	1.4	0.96	3.83
30 cm, *ρ* = 0.9 g∙cm^−3^	310	19.3	1.1	0.98	2.65
30 cm, *ρ* = 0.5 g∙cm^−3^	300	32.7	1.6	0.98	1.47
20 cm, *ρ* = 1.3 g∙cm^−3^	285	21.9	1.3	0.98	2.55
20 cm, *ρ* = 0.9 g∙cm^−3^	294	26.9	1.3	0.99	1.77
20 cm, *ρ* = 0.5 g∙cm^−3^	242	75.3	1.1	1.00	0.98
10 cm, *ρ* = 1.3 g∙cm^−3^	272	63.4	1.3	1.00	1.28
10 cm, *ρ* = 0.9 g∙cm^−3^	163	126.6	2.7	1.00	0.88
10 cm, *ρ* = 0.5 g∙cm^−3^	185	153.8	7.6	0.99	0.49
Control without load	63	338.5	5.7	1.00	0.00

**Table 2:** Results of the Dunnett’s C test (significance level 0.05).

Test Group Versus Control	*p*-Value	Statistically Significant
30 cm, *ρ* = 1.3 g∙cm^−3^	0.00003	yes
30 cm, *ρ* = 0.9 g∙cm^−3^	0.00003	yes
30 cm, *ρ* = 0.5 g∙cm^−3^	0.00005	yes
20 cm, *ρ* = 1.3 g∙cm^−3^	0.00004	yes
20 cm, *ρ* = 0.9 g∙cm^−3^	0.00004	yes
20 cm, *ρ* = 0.5 g∙cm^−3^	0.00036	yes
10 cm, *ρ* = 1.3 g∙cm^−3^	0.00019	yes
10 cm, *ρ* = 0.9 g∙cm^−3^	0.00685	yes
10 cm, *ρ* = 0.5 g∙cm^−3^	0.01790	yes

**Table 3:** Comparison of results of superabsorbent polymer usage as a soil additive.

Authors	Applied Superabsorbent Polymer Additions (% *w/w*)	Type of Superabsorbent Polymer	Type of Soil	Bulk Density (g∙cm^−3^)	Volume Moisture (% *v/v*)	Mass Moisture (% *w/w*)
Shahid et al. [54]	0	poly(Acrylamide-*co*-acrylic acid)/AlZnFe_2_O_4_	sandy loam	1.46		25
	0.1					34
	0.2					36
	0.3					45
	0.4					55
Leciejewski [52]	0	Super Absorbent Plus	loamy sand	1.67	26	
	0.2				30	
	0.3				32	
Baran et al. [58]	0	Agroaquagel	loamy sand	1.29	32	
	0.2				41	
	0.6				45	
	1				55	
Hutterman et al. [15]	0	N/a	sandy soil	N/a		22
	0.4					33
Narjary et al. [57]	0	cellulose based grafted and crosslinked anionic polyacrylate	sandy soil	1.6	24	
	0.5				32	
	0.7				38	
Akhter et al. [53]	0	Polymerisation of acrylamide (*N*,*N*-methylbis-acrylamide) and mixed Na and K salts of acrylic acid	sandy loam	N/a		28
	0.1					34
	0.2					36
	0.3					42
